# High levels of circulating miR-19a-3p in patients with metastatic HER2 + breast cancer are associated with a favorable prognosis and anti-tumor immune responses

**DOI:** 10.1186/s13058-025-02174-8

**Published:** 2026-01-26

**Authors:** Evan N. Cohen, Hui Gao, Sanda Tin, Qiong Wu, Cristina Ivan, Naoto T. Ueno, Wendy A. Woodward, James M. Reuben, Simone Anfossi

**Affiliations:** 1 Present Address: Department of Translational Molecular Pathology, The University of Texas MD Anderson Cancer Center, Houston, TX USA; 2 Department of Hemopathology, The University of Texas MD Anderson Cancer Center, Houston, TX USA; 3 Present Address: Department of Breast Medical Oncology, The University of Texas MD Anderson Cancer Center, Houston, TX USA; 4 Present Address: Department of Thoracic Radiation Oncology, The University of Texas MD Anderson Cancer Center, Houston, TX, USA; 5 Present Address: Caris Life Science, Irving, TX 75039 USA; 6 Department of Experimental Therapeutics, The University of Texas MD Anderson Cancer Center, Houston, TX USA; 7 Center for RNA Interference and Non-Coding RNAs, The University of Texas MD Anderson Cancer Center, Houston, TX USA; 8 Present Address: Translational and Clinical Research Program, University of Hawaiʻi Cancer Center, Honolulu, HI USA; 9 Present Address: Department of Breast Radiation Oncology, The University of Texas MD Anderson Cancer Center, Houston, TX USA; 10 Present Address: The Morgan Welch IBC Clinic and Research Program, The University of Texas MD Anderson Cancer Center, Houston, TX USA; 11 Immunology Program, The University of Texas MD Anderson Cancer Center UTHealth Houston Graduate School of Biomedical Sciences, The University of Texas MD Anderson Cancer Center, Houston, TX USA

**Keywords:** Trastuzumab, ADCC, NK cells, CD4 + Th1 cells, miR-19a-3p, HER2 + metastatic breast cancer, Anti-tumor immune response

## Abstract

**Background:**

Trastuzumab, combined with chemotherapy, is the current standard treatment for both metastatic and early-stage HER2-positive (HER2 +) breast cancer. One of the mechanisms of action of trastuzumab is antibody-dependent cellular cytotoxicity (ADCC), which involves engaging FcγRIIIA (CD16) on natural killer (NK) cells. A competent immune system and properly functioning NK cells are crucial for effective ADCC, as they can influence favorable clinical outcomes. Resistance to trastuzumab often develops after about one year. We previously reported that elevated levels of miR-19a-3p in the serum of patients with metastatic HER2 + breast cancer treated with trastuzumab were associated with a favorable prognosis. Here, we aim to identify the mechanism and the immune cells responsible for elevated serum levels of miR-19a-3p.

**Methods:**

Peripheral blood mononuclear cells (PBMCs) from healthy individuals were used to isolate naïve CD4 + T cells and NK cells. Naïve CD4 + T cells were polarized into CD4 + Th1 and CD4 + Th2 cells. NK cells were utilized for the ADCC assay. Levels of transcription factors, cytokines, and miR-19a-3p were measured using RT-qPCR. Surface markers and cytokines were analyzed by flow cytometry to characterize immune cell phenotypes.

**Results:**

In vitro NK cell-mediated ADCC resulted in increased levels of miR-19a-3p released into the supernatants after killing breast cancer cells. In vitro polarized CD4 + Th1 cells expressed and secreted higher levels of miR-19a-3p than CD4 + Th2 cells. Over a long-term in vitro culture (24 days), anti-CD3/CD28 restimulation sustained higher levels of miR-19a-3p in CD4 + Th1 cells compared to CD4 + Th2 cells and their respective supernatants. CD4 + Th1 cells developed a central memory T (T_CM_) phenotype (CD45RO + CCR7 + CD62L +) and expressed and secreted higher levels of miR-19a-3p than CD4 + Th2 cells. In patients with HER2 + metastatic breast cancer, those with elevated serum levels of miR-19a-3p and a favorable prognosis had a larger percentage of circulating activated T cells and NK cells in their blood compared to patients with lower serum levels of miR-19a-3p and a poor prognosis. The small cohort (n = 15) limits the statistical power of our retrospective study.

**Discussion:**

Our findings suggest that elevated levels of miR-19a-3p in the serum of patients with HER2 + metastatic breast cancer may result from effective NK cell-mediated ADCC and activation of CD4 + Th1 cells, which could be responsible for the anti-tumor immune response associated with a favorable prognosis. Blood levels of miR-19a-3p might help identify breast cancer patients who have effective trastuzumab-induced anti-tumor immune responses.

**Supplementary Information:**

The online version contains supplementary material available at 10.1186/s13058-025-02174-8.

## Introduction

Breast cancer is the most commonly diagnosed cancer and the second leading cause of cancer-related death among women, with an estimated 297,790 new cases (32%) and 42,170 estimated deaths (14%) in 2025 [1]. Although the mortality rate has steadily declined over the past three decades due to secondary prevention and advances in treatment, the incidence of breast cancer has gradually increased by 0.5% per year since the mid-2000s [2]. HER2 is overexpressed in approximately 15–20% of human breast cancers, and HER2 amplification is associated with poor survival in patients with early-stage and metastatic breast cancer [3]. Trastuzumab (Herceptin), when combined with chemotherapy, can extend the survival of patients with early stage and metastatic HER2 overexpressed (> 30% of tumor cells, 3 + by immunohistochemistry, IHC) and/or amplified (FISH HER2/CEP7 ratio > 2.2) breast cancer [4, 5]. By binding to the extracellular domain IV (ECD IV) of HER2, trastuzumab suppresses intracellular HER2 signaling pathways, inhibits cell cycle progression, and mediates antibody-dependent cell-mediated cytotoxicity (ADCC) [6]. Part of trastuzumab's effectiveness depends on ADCC, where the fragment crystallizable (Fc) region of trastuzumab interacts with FcγRIIIA (CD16) on natural killer (NK) cells, leading to NK cell activation, cytokine production, and tumor cell lysis through perforin/granzyme B-mediated killing [7–10]. In vivo studies have demonstrated that both innate and adaptive immune cells are essential for the anti-tumor activity of trastuzumab, involving NK cells, CD8αα + dendritic cells (DCs), adaptive CD8αβ + T cells, Type 1 CD4 + T helper (CD4 + Th1), and type I and II interferons as key players [11, 12]. Chemotherapy combined with anti-HER2 antibodies can increase tumor infiltration and enhance the activity of NK cells and lymphocytes [13–16], thereby improving the therapeutic effect. Reports indicated that the release of endogenous danger signals, such as high-mobility-group box 1 (HMGB1), caused by chemotherapy-mediated tumor cell death, can stimulate DC cross-priming in a TLR-4/MyD88-dependent manner [17] and induce robust innate immune responses and antibody-mediated tumor regression [12]. CD4 + Th1 cells play a crucial role in regulating anti-tumor immunity. IFN-γ and IL-2, secreted by CD4 + Th1 cells, trigger the activation of DCs and enhance the cross-presentation of tumor-associated antigens (TAAs), thereby priming anti-tumor CD8 + T cells and supporting their differentiation into mature CD8 + cytotoxic T lymphocytes (CTLs). This process promotes CD8 + CTL proliferation and anti-tumor cytotoxic activity [18–20], and it enhances the cytotoxic response of NK cells [21]. In patients with HER2 + breast cancer, elevated levels of anti-HER2 CD4 + T-bet + IFN-γ + Th1 cells correlate with pathologic responses following neoadjuvant trastuzumab and chemotherapy, and they are associated with a reduced risk of recurrence and improved prognosis [16].

The introduction of HER2-targeted therapies, such as trastuzumab, pertuzumab, and antibody–drug conjugates, including trastuzumab deruxtecan (T-DXd) and trastuzumab emtansine (T-DM1) [7, 22], has greatly improved prognosis. However, the responses to these therapies, especially when combined with chemotherapy, vary widely, ranging from complete response to no benefit [23, 24]. Approximately 15–23% of patients with early-stage disease develop recurrent disease [4], whereas de novo and acquired resistance mainly occur in the metastatic setting [25]. To date, aside from HER2 expression status and genetic biomarkers, no reliable predictive blood biomarker for anti-HER2 therapies has been validated for clinical use in patients with HER2-positive breast cancer treated with trastuzumab [26–28]. The presence of immune cell infiltration is associated with a more favorable response to anti-HER2 therapies [29–32]. This heterogeneity in treatment response and resistance development necessitates the identification of biomarkers to determine which patients are likely to benefit from treatment.

MicroRNAs (miRNAs) are short non-coding RNAs (ncRNAs) that measure approximately 21 ribonucleotides in length and regulate the expression of protein-coding genes [33–35]. Deregulated levels of miRNAs are associated with various types of cancer [33]. MiRNAs can also be found in the blood (serum and plasma) of cancer patients, as circulating miRNAs and their altered levels may indicate the clinical progression of the disease and serve as blood tumor biomarkers [36]. MiR-19a-3p is part of the miR-17–92 cluster, which includes six miRNAs: miR-17, miR-18a, miR-19a, miR-19b, miR-20a, and miR-92a. These miRNAs play a central regulatory role in immune cell development, function, and also in diseases, including cancer, when deregulated [37–39]. In particular, T cell receptor (TCR)-mediated activation of the mTOR signaling pathway induces MYC-mediated transcription of the miR-17–92 cluster, promoting T cell survival and proliferation by targeting the pro-apoptotic Bim and PTEN [37, 40, 41].

In our previous study, we reported that elevated serum levels of miR-19a-3p, as measured at the beginning of the study (LAB08-0199), were associated with a favorable prognosis for patients with metastatic HER2 + breast cancer treated with trastuzumab [42]. In the current study, we aimed to understand the source of these elevated levels of miR-19a-3p and the underlying mechanism. We discovered that miR-19a-3p is expressed at high levels in breast cancer tissue, particularly in HER2 + and triple-negative breast cancer (TNBC) subtypes, compared to the estrogen receptor-positive (ER +) subtype. Furthermore, NK cell-mediated ADCC resulted in higher levels of miR-19a-3p released from killed cells. Additionally, activated CD4 + Th1 cells produced and secreted higher levels of miR-19a-3p than CD4 + Th2 cells in vitro.

NK cell-mediated ADCC (perforin/granzyme-induced apoptosis) can increase tumor cell membrane permeability by forming pores [43] and trigger CD4 + Th1-mediated antitumor immunity [44, 45]. In turn, CD4 + Th1 cells secrete high levels of IFN-γ and IL-2, which are critical for activating the antitumor activity of CD8 + CTLs [46] and NK cells [47]. Therefore, we hypothesized that in patients with metastatic HER2 + breast cancer treated with trastuzumab, the elevated levels of miR-19a-3p, associated with a better prognosis, may result from passive release from apoptotic or dead tumor cells killed by NK cell-mediated ADCC [45] and the effective activation of CD4 + Th1 cells. The combination of these two effects may partly explain the association between high levels of miR-19a-3p and a favorable prognosis in patients with metastatic HER2 + breast cancer treated with trastuzumab. Elevated serum levels of miR-19a-3p in patients with metastatic HER2 + breast cancer treated with trastuzumab may serve as a tumor biomarker for identifying those with effective immune responses who are likely to benefit from anti-HER2 therapy.

## Materials and Methods

### Patient cohort

This retrospective study represents a continuation of our previous research on circulating miRNAs in the serum of breast cancer patients [42], where the blood samples from patients with HER2 + metastatic breast cancer were collected at the beginning of the study, before starting a new line of therapy. We investigated the phenotype of immune cells from blood samples of patients enrolled in our previous study conducted under the laboratory protocol LAB08-0199 at The University of Texas MD Anderson Cancer Center from October 2008 to May 2010. The study has been approved by the Institutional Review Board (IRB) at The University of Texas MD Anderson Cancer Center and adhered to the tenets of the Declaration of Helsinki. Written informed consent was obtained from each participant before sample collection. We gathered immune cell profiling data from a total of 15 patients with metastatic HER2 + breast cancer, including those with high serum levels of miR-19a-3p (good prognosis) and those with low serum levels of miR-19a-3p (poor prognosis). The levels of miR-19a-3p values and the categories miR-19a-3p^high^ and miR-19a-3p^low^ originate from our previous work [42]. Patients' clinical and histopathological information is summarized in Table 1.

**Table 1:** Clinical characteristics of patients with metastatic HER2 + breast cancer

	Age	IBC	Stage	Race	ER	PR	HER2	Histology	Grade	Bone mets	miR-19a-3p	
1	76	IBC	IV	WHITE	ER +	PR-	HER2 +	IDC	3	0	0.20	Low
2	69	non-IBC	IV	BLACK	ER +	PR +	HER2 +	IDC	3	0	0.35	Low
3	53	non-IBC	IV	HISPANIC	ER-	PR-	HER2 +	IDC	3	0	0.52	Low
4	44	IBC	IV	WHITE	ER-	PR-	HER2 +	IDC	3	1	0.66	Low
5	54	non-IBC	IV	WHITE	ER +	PR +	HER2 +	IDC	2	1	0.66	Low
6	40	IBC	IV	HISPANIC	ER-	PR +	HER2 +	N/A	3	1	0.72	Low
7	53	non-IBC	IV	WHITE	ER-	PR-	HER2 +	IDC	3	1	0.91	Low
8	56	non-IBC	IV	WHITE	ER-	PR-	HER2 +	IDC	3	1	1.32	High
9	62	IBC	IV	WHITE	ER +	PR +	HER2 +	IDC	3	0	1.57	High
10	55	IBC	IV	WHITE	ER-	PR-	HER2 +	IDC	2	1	1.71	High
11	38	non-IBC	IV	WHITE	ER +	PR-	HER2 +	IDC	2	1	1.89	High
12	56	IBC	IV	WHITE	ER +	PR-	HER2 +	IDC	3	0	2.46	High
13	38	IBC	IV	WHITE	ER-	PR-	HER2 +	IDC	3	0	2.46	High
14	39	IBC	IV	WHITE	ER +	PR-	HER2 +	MIXED	2	1	2.46	High
15	48	non-IBC	IV	WHITE	ER-	PR-	HER2 +	IDC	3	0	4.04	High

IDC = Invasive Ductal Carcinoma, IBC = Inflammatory breast Cancer, ER = Estrogen Receptor, PR = Progesteron Receptor

High/low cut-off = mean levels of miR-19a-3p in the serum normal donors (n = 30) plus 2 standard deviations

### miR-19a-3p expression in breast cancer subtypes (TCGA)

The breast cohort used in this study is the Breast Invasive Carcinoma TCGA Pan-Cancer (PANCAN) cohort. The PAM50 subtypes were obtained from cBioPortal (https://www.cbioportal.org/). The expression data for hsa-miR-19a-3p (MIMAT0000073) were obtained from the UCSC XENA repository (https://xenabrowser.net/datapages/), specifically from the TCGA Pan-Cancer (PANCAN) cohort, using the miRNA mature strand expression dataset with batch effect-normalized miRNA data log2(norm_value + 1) [48–50]. Box and whisker plots were generated in R version 4.4, where the box encompasses the 25th percentile, median, and 75th percentile, and the whiskers extend to 1.5 times the interquartile range. These plots display miRNA expression across PAM50 subtypes. The displayed p-value was generated using the nonparametric Kruskal–Wallis test for multiple group comparisons. Post hoc Dunn test p-values were also generated in R and presented in Table 2.

**Table 2:** *P* values of comparison among the different groups (Post hoc Dunn test)

Expression	p value	expr_Basal	expr_Her2	expr_LumA	expr_LumB	expr_Normal	expr_PAM50Normal	BasalvsHer2	BasalvsLumA	BasalvsLumB	BasalvsNormal
miR-19a-3p	1.90008479164359e-67	5.99	4.39	3.95	4.72	3.6	4.56	1.70015523564339e-11	3.7031295573817e-60	3.44540213457777e-16	1.98728435035002e-40

Expression	BasalvsPAM50_Normal	Her2vsLumA	Her2vsLumB	Her2vsNormal	Her2vsPAM50_Normal	LumAvsLumB	LumAvsNormal	LumAvsPAM50_Normal	LumBvsNormal	LumBvsPAM50_Normal	NormalvsPAM50_Normal
miR-19a-3p	1.37450698487296e-08	0.000239225	1	2.64237917216115e-06	1	6.65752744980216e-11	0.064371508	0.121593907	9.74387986552627e-11	0.729416303	0.005027661

Comparison: Basal vs Her2 vs LumA vs LumB vs Normal vs PAM50_Normal

Method: Kruskal–Wallis

### Breast cancer cell lines

A panel of human breast cancer cell lines was used in this study. MCF-7 (ER +) and SKBR3 (HER2 +) cells were obtained from the American Type Culture Collection (Manassas, VA, USA), and KPL-4 (HER2 +) cells were provided courtesy of Dr. Junichi Kurebayashi (Kawasaki Medical School, Kurashiki, Japan) [51]. Breast cancer cells were cultured in DMEM/F-12 medium supplemented with 10% fetal bovine serum (FBS) (Tissue Culture Biologicals, Seal Beach, CA, USA). The measurements of miR-19a-3p in the cells and their supernatants were performed in three experimental replicates and three technical replicates.

### NK cell isolation and CD4 + Th1 and CD4 + Th2 cell polarization

Peripheral blood mononuclear cells (PBMCs) were collected from normal individuals’ blood to isolate NK cells for in vitro ADCC assay and naïve CD4 + T cells for polarization into CD4 + Th1 and CD4 + Th2 cells. Briefly, 40–50 mL of blood was collected in heparin tubes, diluted 1:1 with PBS, and layered onto a density gradient medium (Ficoll-Paque PLUS™; GE Healthcare Life Sciences, Little Chalfont, UK). The samples were then centrifuged (400 × g for 30 min at 25 °C) to isolate PBMCs. NK cells were isolated from PBMCs by magnetic bead sorting using the NK Cell Isolation Kit, Human (Miltenyi Biotec, Inc., Auburn, CA, USA) starting from 80–90 × 10^6^ PBMCs. The purity of NK cells (> 95%) was evaluated by flow cytometry using PE mouse anti-human CD56 and FITC mouse anti-human CD16 (BD Pharmingen™, Franklin Lakes, NJ, USA). NK cells were cultured in RPMI 1640 medium supplemented with 10% FBS (Tissue Culture Biologicals, Seal Beach, CA, USA) overnight before the ADCC assay.

Naïve T cells (CD4 + CD45RA + CD62L +) were isolated from PBMCs using Fluorescence-Activated Cell Sorting (FACS) using BD FACSAria II Cell Sorter starting from 80–90 × 10^6^ PBMCs at North Campus Flow Cytometry and Cellular Imaging Core Facility (The University of Texas MD Anderson Cancer Center). Gating strategy is detailed in Supplementary Fig. 1A. PBMCs were stained with FITC mouse anti-human CD45RA, APC mouse anti-human CD4, and PE mouse anti-human CD62L (BD Pharmingen™, Franklin Lakes, NJ, USA). Then, around 1 × 10^6^ of naïve CD4 + CD45RA + CD62L + T cells were activated through T-cell receptor (TCR) stimulation using a 24-well plate (Costar, Cambridge, MA, USA) coated with 8 µg/mL per well of anti-human CD3 (Clone: UCHT1; Coulter Immunology, Hialeah, FL, USA) and 2 µg/mL per well of anti-human CD28 (BD Biosciences, San Jose, CA, USA) in RPMI 1640 supplemented with 10% FBS containing 20 IU/mL of rhIL-2 (Miltenyi Biotec, Inc., Auburn, CA, USA). Two separated cell culture conditions were used, each with specific cytokine/antibody cocktails to polarize activated CD4 + T cells into CD4 + Th1 and CD4 + Th2 cells. Specifically, 2 ng/mL of rhIL-12 (BD Pharmingen™, Franklin Lakes, NJ, USA) and 100 ng/mL of rhIFN-γ (R&D Systems, Inc., Minneapolis, MN, USA) plus 1 µg/mL of anti-hIL-4 (BD Pharmingen™, Franklin Lakes, NJ, USA) were added for CD4 + Th1 cell polarization, while 1000 IU/mL of rhIL-4 (R&D Systems, Inc., Minneapolis, MN, USA) plus 2 ng/mL of anti-hIL-12 and 5 µg/mL of anti-hIFN-γ (BD Pharmingen™, Franklin Lakes, NJ, USA) were added for CD4 + Th2 cell polarization. Activated CD4 + T cells under polarizing conditions were cultured at 37 °C for 3–4 days. Afterward, they were collected, washed, plated back into an uncoated 24-well plate, and cultured for an additional 3–4 days in RPMI supplemented with 10% FBS containing 20 IU/mL of rhIL-2 and the respective cytokine/antibody cocktails to complete the polarization (a total of seven to eight days). Cytokine/antibody cocktails were not added to activated CD4 + T cells to generate non-polarized Th0 cells. The polarization of CD4 + Th1 and CD4 + Th2 cells was assessed by measuring standard markers, including transcription factors (T-bet and GATA-3), using RT-qPCR, and cytokines (IFN-γ and IL-4), using both flow cytometry and RT-qPCR. We measured the expression of T-bet and IFN-γ for CD4 + Th1 cells and GATA-3 and IL-4 for CD4 + Th2 cells. Both polarized (Th1 and Th2) and non-polarized (Th0) CD4 + T cells were maintained in RPMI 1640 supplemented with 10% FBS with 50 IU/mL of rhIL-2 and restimulated with anti-human CD3-coated 24-well plate (8 µg/mL per well) and anti-human CD28 (2 µg/mL per well) approximately every 7 days to maintain their viability until the end of cell culture, around 24 days in total.

### Flow cytometry and intracellular staining of CD4 + Th1 and CD4 + Th2 cell phenotype measurement

The expression levels of HER2 were measured in MCF-7, SKBR3, and KPL-4 cells by flow cytometry using 1 µg/mL of trastuzumab (Herceptin, Genentech, Inc., from The University of Texas MD Anderson Cancer Center Pharmacy) for around 1 × 10^6^ cells. FITC mouse anti-human IgG (BD Pharmingen™, Franklin Lakes, NJ, USA) was used as a secondary labeled antibody. FITC mouse anti-human IgG alone was used as an isotype control. The PE mouse anti-human HER2/neu (BD Pharmingen™, Franklin Lakes, NJ, USA) was used as a comparison for HER2 expression. Unstained cells were used as a control for autofluorescence levels. PE Mouse Anti-Human CD45RO and PE Mouse Anti-Human CD62L (BD Pharmingen™, Franklin Lakes, NJ, USA) were used to measure the expression of CD45RO and CD62L in CD4 + central memory T (T_CM_) cells. To evaluate the production of IFN-γ and IL-4, CD4 + Th1 and CD4 + Th2 cells were stimulated with 1 µg/mL of ionomycin and 100 ng/mL of phorbol 12-myristate 13-acetate (PMA) for 4 h, with 10 µg/mL of brefeldin A (BFA) added during the last 2 h. T cells were fixed and permeabilized using the BD Cytofix/Cytoperm™ Fixation/Permeabilization Kit (BD Pharmingen™, Franklin Lakes, NJ, USA) and then stained with PE Rat Anti-Human IL-4 and FITC Mouse Anti-Human IFN-γ (BD Pharmingen™, Franklin Lakes, NJ, USA) for intracellular cytokine staining. PE Mouse IgG1, κ Isotype Control and FITC Mouse IgG1, κ Isotype Control (BD Pharmingen™, Franklin Lakes, NJ, USA), or unstained cells were used as an isotype control. A similar method was used for Th0 CD4 + T cells, where FITC Rat Anti-Human IL-2 and PE Rat Anti-Human IL-13 were used as additional markers (BD Pharmingen™, Franklin Lakes, NJ, USA). Gating strategy is detailed in Supplementary Fig. 1B. A 1-h stimulation with ionomycin/PMA was performed before RNA extraction for the measurement of IFN-γ and IL-4 by RT-qPCR.

Breast cancer patients’ PBMCs were investigated for CD4 + Th1, CD4 + Th2, and NK cells using the same methods as those for normal individuals’ PBMCs described above, and as previously reported [52]. Antibodies from BD Pharmingen™ used in this analysis included: APC-Cy™7 Mouse Anti-Human CD45; PE-Cy™7 Mouse Anti-Human CD3; V450 Mouse Anti-Human CD45; PE-Cy™7 Mouse Anti-Human CD4; APC Mouse Anti-Human CD56; PE Mouse Anti-Human CD16; PE Rat Anti-Human IL-4; APC Mouse Anti-Human IFN-γ; FITC Rat Anti-Human IL-2; PerCP-Cy™5.5 Mouse Anti-Human IL-4. Gating strategy is detailed in Supplementary Fig. 1C. All flow cytometry was performed using the FACSCalibur flow cytometer (Becton Dickinson, Franklin Lakes, NJ, USA) and the CellQuest software, and data analysis was conducted using FlowJo™ Software (Becton Dickinson, Franklin Lakes, NJ, USA).

## ADCC assay

Approximately 30,000 cells/well of breast cancer cells were plated in 96-well U-shaped-bottom plates (Costar, Cambridge, MA, USA) in RPMI 1640 medium supplemented with 10% FBS. Wells with only breast cancer cells (Cntr) and breast cancer cells combined with trastuzumab (+ Trast) were used as controls for basal secretion of miR-19a-3p. Freshly sorted and overnight cultured NK cells were added at various NK:breast cancer cell ratios (10:1, 5:1, 1:1) to assess the basal NK cell killing activity. Trastuzumab was added to induce NK cell-mediated ADCC in wells where NK cells were added at different NK:breast cancer cell ratios (10:1 + T, 5:1 + T, 1:1 + T). A 50 µg/ml concentration of trastuzumab was used, as it is similar to the concentration at the plateau in the blood of breast cancer patients [53]. The cells were incubated at 37 °C for 4 h. The cells and respective supernatants from each well were collected to evaluate the basal NK cell killing, NK cell-mediated ADCC, and the levels of released miR-19a-3p. Basal NK cell killing and NK cell-mediated ADCC (10:1 NK:breast cancer cell ratio) were measured using the FITC Annexin V Apoptosis Detection Kit (BD Pharmingen™, Franklin Lakes, NJ, USA) in combination with Propidium Iodide (PI) (ThermoFisher Scientific, Cambridge, MA, USA) and analyzed by flow cytometry. The size-exclusion gating strategy excluded NK cells from breast cancer cells. The supernatants were centrifuged at high speed (3,000 × g) to remove cell debris and then stored at -80 °C before RNA extraction and measurement of miR-19a-3p. The basal NK cell killing and NK cell-mediated ADCC assay were performed in two experimental replicates and three technical replicates.

## RNA extraction and RT-qPCR

Total RNA was isolated from cells and supernatants using the Total RNA Purification Kit (Norgen Biotek Corporation, Thorold, ON, Canada). The amount of 20 fmol of cel-miR-248 was spiked-in during the RNA extraction from supernatants to work as a reference for RT-qPCR normalization of miR-19a-3p levels. U6 snRNA and cel-miR-248 were used as references for the RT-qPCR normalization of miR-19a-3p levels in cells and supernatants, respectively. GAPDH was used as a reference for normalizing gene levels in RT-qPCR. The total RNA concentrations were measured using a NanoDrop 2000 spectrophotometer (Thermo Scientific, Wilmington, DE). The RNA of each sample was converted into cDNA using the TaqMan MicroRNA Reverse Transcription kit for miRNAs and the High-Capacity cDNA Reverse Transcription Kit for genes (Thermo Scientific, Wilmington, DE), and RT-qPCR was performed as previously described using TaqMan Assay (Thermo Scientific, Wilmington, DE) [42]. The levels of miR-19a-3p and the genes were reported as 2^−ΔCt^ values calculated using the formula ΔCt = mean Ct_(target)_ – mean Ct_(reference)_, where Ct = threshold cycle, target = T-bet, GATA-3, IFN-γ, IL-4, miR-19a-3p, and reference = U6 snRNA or cel-miR-248 or GAPDH. The TaqMan Assays used are: miR-19a-3p (Assay ID: 000395); U6 snRNA (Assay ID: 001973); cel-miR-248 (Assay ID: 007218_mat); T-bet (Assay ID: Hs00894392_m1); GATA-3 (Assay ID: Hs00231122_m1); IFN-γ (Assay ID: Hs00989291_m1); IL-4 (Assay ID: Hs00174122_m1); GAPDH (Assay ID: Hs02758991_g1).

### Breast cancer cell transfection with miR-19a-3p mimic and inhibitor

The two breast cancer cell lines, MCF-7 and KPL-4, were transfected with either miR-19a-3p mimic or inhibitor (Thermo Scientific, Wilmington, DE) at a concentration of 80 nM using Lipofectamine 2000 (Invitrogen) according to the manufacturer’s instructions. The RNA was extracted after 48 h and stored at -80 °C before measuring the levels of miR-19a-3p by RT-qPCR.

### Statistical analysis

The Unpaired t-test was used to compare two data groups from in vitro experiments. The Mann—Whitney U test was used to compare the data of the two patient groups. Data analysis was performed using GraphPad Prism 10. P value summary: ns indicates no statistical significance; *p < 0.05; **p < 0.01; ***p < 0.001; ****p < 0.0001.

## Results

### HER2 + breast cancer cells express high levels of miR-19a-3p

**Fig. 1:**
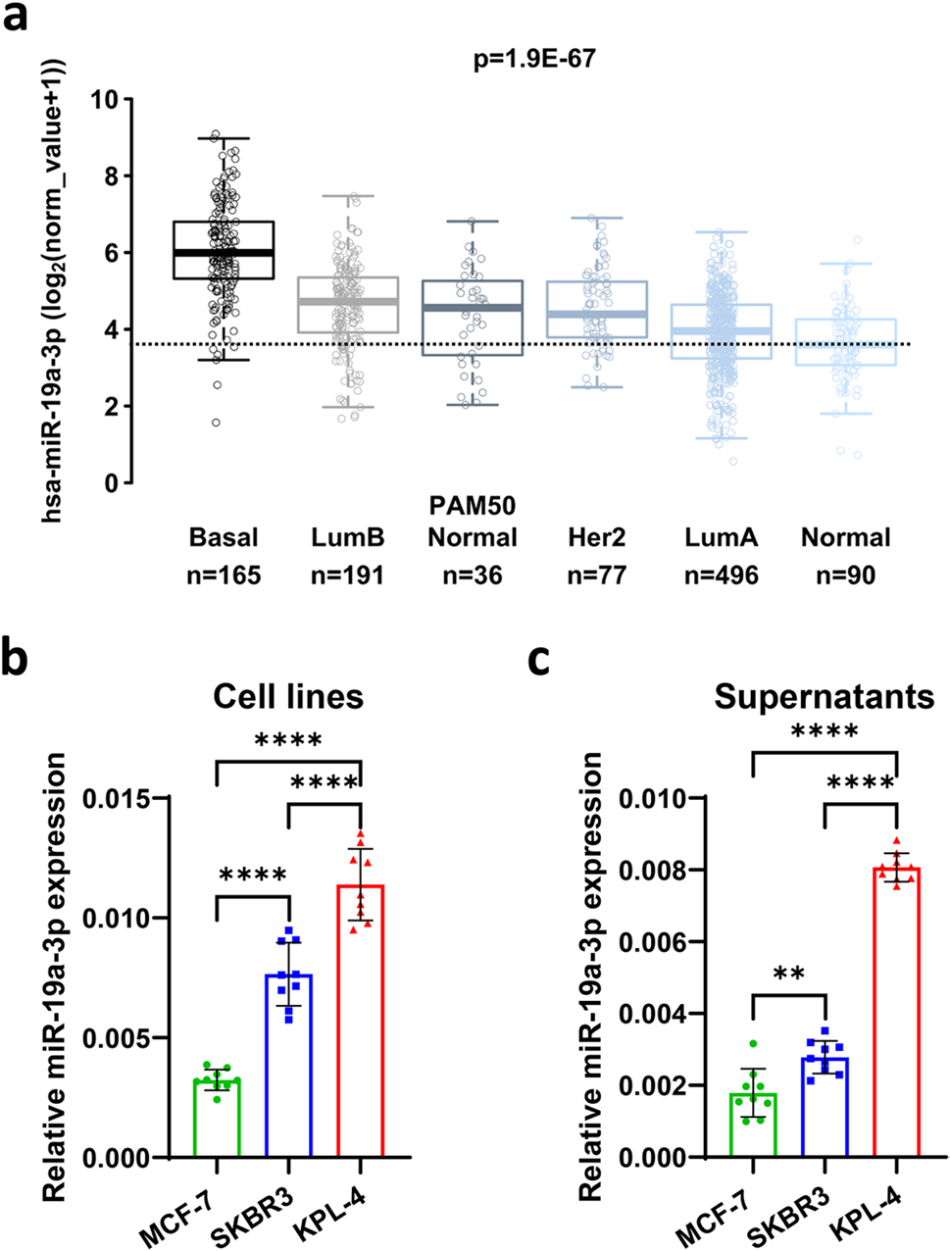
Expression and secretion levels of miR-19a-3p in breast cancer cells. **a)** Breast cancer cells express different levels of miR-19a-3p depending on the subtypes, with TNBC (basal) expressing the highest levels compared to luminal B, PAM50 Normal, HER2+, luminal A, and normal cells (TCGA data). The data are shown as box and whisker plots, where the box encompasses the 25th percentile, median, and 75th percentile, and the whiskers extend to 1.5 times the interquartile range; **b)** the two HER2+ breast cancer cell lines KPL-4 and SKBR3 express higher level of miR-19a-3p than the luminal A MCF-7, consistent with TCGA data; **c)** the three breast cancer cell lines secrete varying amounts of miR-19a-3p, following an expression pattern similar to that in their parental cells. The results are shown as the mean ± SD of three technical replicates from 3 experimental replicates

This study investigated the potential sources that regulate the levels of miR-19a-3p in the serum of patients with metastatic HER2 + breast cancer treated with trastuzumab. We began by analyzing the TCGA database to assess the expression of miR-19a-3p in breast cancer tissue. Our analysis showed that miR-19a-3p was highly expressed in TNBC (basal) and HER2 + breast cancer tissues compared to ER + (luminal A) and normal breast tissue. (Fig. 1A and Table 2). Next, we selected two HER2 + (SKBR3 and KPL-4) and one ER + (MCF-7) breast cancer cell lines to measure the expression of miR-19a-3p. SKBR3 and KPL-4 cells expressed higher levels of miR-19a-3p than MCF-7 cells (Fig. 1B). We then measured the levels of miR-19a-3p in the cell culture supernatants of these cells. The supernatants from KPL-4 and SKBR3 cells contained higher levels of miR-19a-3p than those from MCF-7 cells (Fig. 1C).

In summary, HER2 + breast cancer tissue and cells express high levels of miR-19a-3p, which can be released into the extracellular space by tumor cells and measured.

### NK cell-mediated ADCC increased the levels of miR-19a-3p released into the cell supernatants

**Fig. 2:**
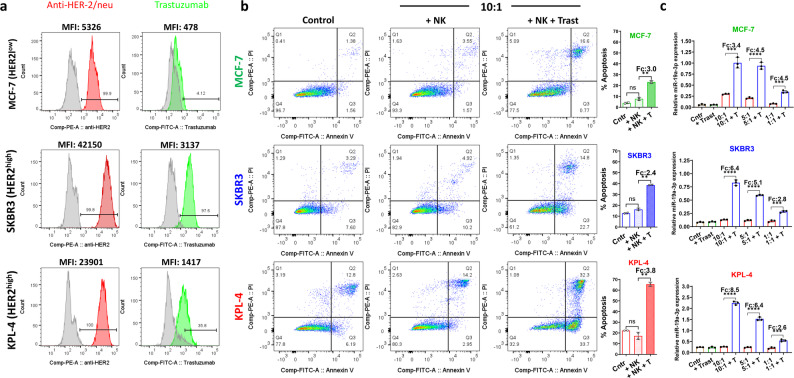
Binding of trastuzumab to breast cancer cells and NK cell-mediated ADCC. **a)** Trastuzumab (Herceptin) binds to breast cancer cells based on HER2 expression (overexpression/amplification) levels showing a similar intensity pattern with the commercial anti-HER2/neu (BD Pharmingen) (MFI = Mean Fluorescence Intensity); **b)** representative dot plot from two experimental replicates shows that trastuzumab significantly increased the NK cell-mediated ADCC of breast cancer cells (NK:breast cancer cell 10:1 ratio). The results are shown as the mean ± SD of 2 technical replicates; **c)** Trastuzumab induced an increase in the levels of miR-19a-3p measured in the supernatants. A decreasing pattern of the levels of miR-19a-3p was measured according to the decreasing NK:breast cancer cell ratios (10:1, 5:1, 1:1). Treatments include: (Cntr = control) tumor cells only; (T = trastuzumab) trastuzumab + tumor cells; (10:1, 5:1, 1:1) NK:breast cancer cell ratios; (10:1 + T, 5:1 + T, 1:1 + T) NK:breast cancer cell ratios + trastuzumab. The results are presented as the mean ± SD of three technical replicates

The combination of chemotherapy and targeted therapy with trastuzumab represents a standard treatment for patients with HER2 + breast cancer [7]. One of the mechanisms of action of trastuzumab is antibody-dependent cell-mediated cytotoxicity (ADCC) [54]. In ADCC, the antibody-mediated activation of NK cells triggers the release of perforin and granzymes by NK cells [8]. The formation of perforin pores can increase tumor cell membrane permeability, causing part of the soluble intracellular content to leak out [43]. Therefore, we investigated in vitro whether NK cell-mediated ADCC triggered by trastuzumab could cause an increased release of miR-19a-3p, which can be detected in the cell supernatants. First, we used flow cytometry to compare the binding levels of trastuzumab to SKBR3 and KPL-4 cells with those to MCF-7 cells, which served as a control. As expected, trastuzumab bound to SKBR3 (MFI: 3137) and KPL-4 (MFI: 1471) cells at higher levels than MCF-7 cells (MFI: 478), showing a pattern similar to that of the commercial mouse anti-human HER2 antibody (Fig. 2A). Differences in the affinity of the two antibodies for the HER2 epitopes and in fluorophore brightness (FITC vs. PE) might explain why trastuzumab shows lower expression levels compared to the commercial mouse anti-human HER2 antibody. In the ADCC assay, NK cells alone did not effectively kill MCF-7, SKBR3, and KPL-4 cells, as there were no significant differences in apoptotic or dead cell levels compared to the control (natural or basal apoptosis level) (Fig. 2B). Trastuzumab significantly enhanced the NK cell-mediated ADCC against SKBR3 cells (about 2.4-fold change) and KPL-4 cells (about 3.8-fold change) (Fig. 2B). Notably, trastuzumab also induced NK cell-mediated ADCC against MCF-7 cells (approximately a threefold increase), even though these cells do not overexpress HER2, and this finding is consistent with a previous report [55]. Next, we evaluated whether NK cell-mediated ADCC could cause an increase in the levels of miR-19a-3p released by apoptotic or dead cells into the supernatants. We observed that enhanced NK cell-mediated ADCC was accompanied by increased levels of miR-19a-3p, as measured in all the breast cancer cell supernatants. As expected, the two HER2 + cells SKBR3 and KPK-4 showed the most significant increase in levels of miR-19a-3p in the supernatants following NK cell-mediated ADCC. A decreasing pattern in levels of miR-19a-3p in the supernatants was observed, consistent with the decreasing NK:breast cancer cell ratios (10:1, 5:1, 1:1) across all three breast cancer cell lines. Additionally, the basal levels of secreted miR-19a-3p by tumor cells (Cntr and + Trast) were much less than those induced by NK cell-mediated ADCC. (Fig. 2C). Finally, we aimed to determine whether the release of miR-19a-3p into the supernatant after NK cell-mediated ADCC was proportional to the cellular level of miR-19a-3p in the breast cancer cells. We transfected MCF-7 cells (which have the lowest miR-19a-3p expression) with a miR-19a-3p mimic and KPL-4 cells (which have the highest miR-19a-3p expression) with a miR-19a-3p inhibitor to respectively increase and decrease the cellular levels of miR-19a-3p (Supplementary Fig. 2A). The levels of miR-19a-3p measured in the supernatants of MCF-7 and KPL-4 cells transfected with the scramble control showed a pattern similar to that in Fig. 2C. The overexpression of miR-19a-3p in MCF-7 cells resulted in a remarkably higher spontaneous release (Cntr and + T) of miR-19a-3p into the supernatants compared to the scramble control. Trastuzumab enhanced NK cell-mediated ADCC and the subsequent release of miR-19a-3p into the supernatant. The levels of miR-19a-3p were much higher in the supernatants of miR-19a-3p mimic-transfected MCF-7 cells than in those of scramble-transfected MCF-7 cells (Supplementary Fig. 2B left). Conversely, downregulating miR-19a-3p in KPL-4 cells resulted in reduced levels of miR-19a-3p spontaneously released into the supernatants (Cntr and + T) compared to the scramble control. Once again, trastuzumab enhanced NK cell-mediated ADCC and the subsequent release of miR-19a-3p into the supernatant. The levels of miR-19a-3p were much lower in the supernatants of miR-19a-3p inhibitor-transfected KPL-4 cells compared to those of scramble-transfected KPL-4 cells (Supplementary Fig. 2B right).

In summary, effective NK cell-mediated ADCC increased the release of miR-19a-3p from apoptotic or dead breast cancer cells, which was much higher than the amount spontaneously secreted by tumor cells. This release of miR-19a-3p was proportional to the cellular levels of miR-19a-3p. Trastuzumab also induced measurable NK cell-mediated ADCC of non-HER2-overexpressing MCF-7 cells.

### T helper type-1 cells expressed and secreted higher levels of miR-19a-3p than T helper type-2 cells

Besides directly killing tumor cells, NK cells also play a crucial role in modulating the anti-tumor response within the tumor microenvironment (TME) by releasing cytokines and chemokines, thus affecting the functions of various immune cells [56]. Specifically, the engagement of trastuzumab with CD16 induces NK cells to secrete IFN-γ [9] and T cell–recruiting chemokines (IL-8, RANTES, MIP-1α, MIP-1β, MCP-1, MDC) [10, 57], contributing to the modulation of anti-tumor immune responses in the TME. Because trastuzumab can enhance CD4 + Th1 cell-regulated anti-tumor immune responses [16, 58], we investigated whether activated CD4 + Th1 cells could produce and secrete miR-19a-3p, thereby contributing to the increased levels of miR-19a-3p in the serum of patients with metastatic HER2 + breast cancer and a favorable prognosis.

**Fig. 3:**
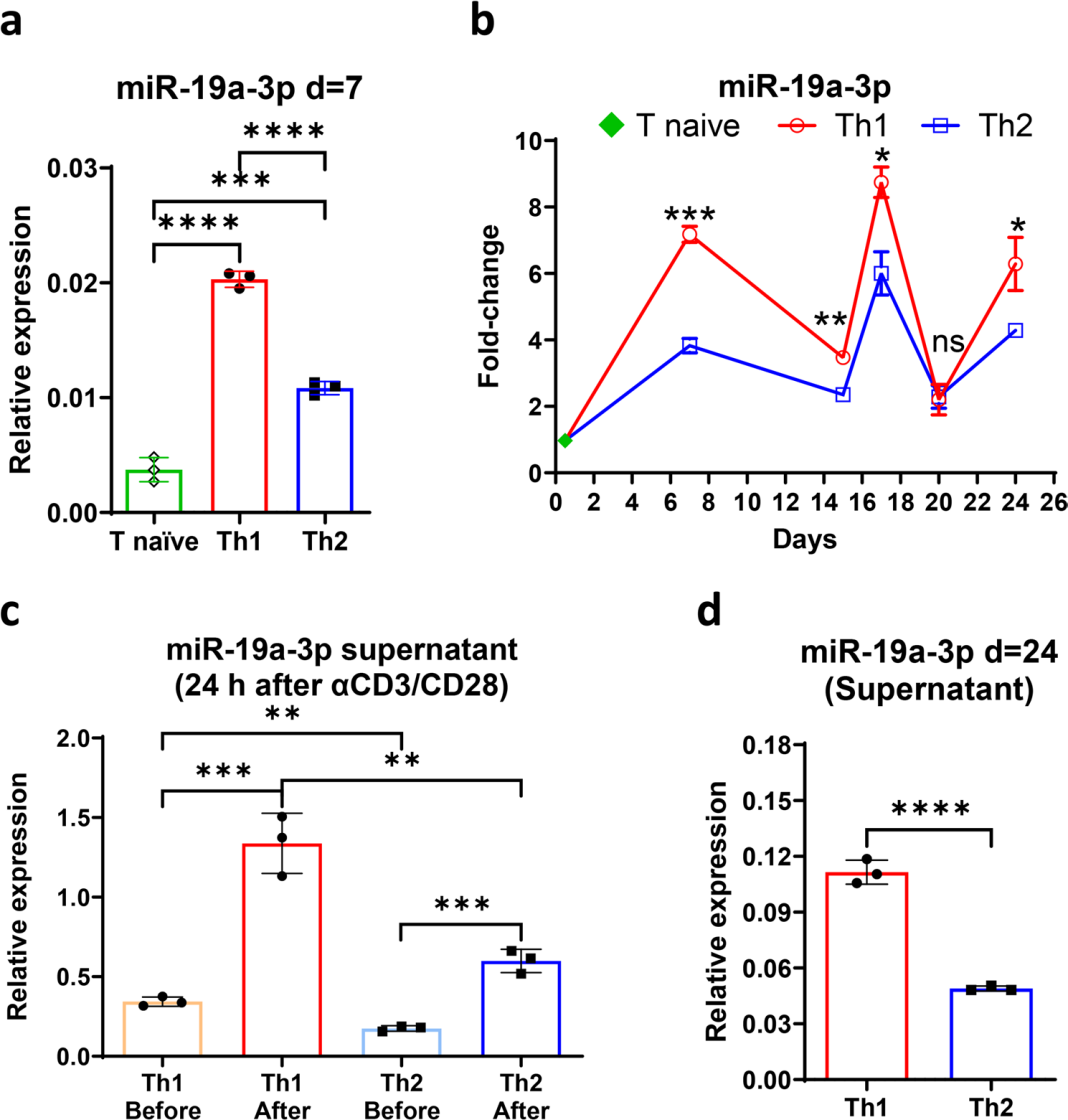
Cellular and secreted levels of miR-19a-3p in CD4+ Th1 and CD4+ Th2 cells. **a) ** Expression levels of miR-19a-3p increased from naïve CD4+ T cells to CD4+ Th1 and CD4+ Th2 cells after seven days of culture, with CD4+ Th1 cells expressing higher levels of miR-19a-3p than CD4+ Th2; **b)** levels of miR-19a-3p varied over time and increased in CD4+ Th1 and CD4+ Th2 cells after restimulation with anti-CD3/CD28 (d=15 and d=20); **c)** CD4+ Th1 cells had higher levels of secreted miR-19a-3p than CD4+ Th2, 24 h after anti-CD3/CD28 restimulation; **d)** levels of miR-19a-3p in the supernatant of CD4+ Th1 and CD4+ Th2 cells were measured at d=24 after anti-CD3/CD28 restimulation at d=20. The results are shown as the mean ± SD of 3 technical replicates. Note: In **c** and **d**, the levels of miR-19a-3p in the supernatants were normalized by the number of CD4+ Th1 and CD4+ Th2 cells in each well

We isolated naïve CD4 + T cells (CD4 + CD45RA + CD62L +) from the peripheral blood of normal individuals through cell sorting and polarized them into CD4 + Th1 and CD4 + Th2 cells (Supplementary Fig. 3A and B). Activation with anti-CD3/CD28 and polarization induced increased levels of miR-19a-3p in both CD4 + Th1 and CD4 + Th2 cells compared to naïve CD4 + T cells. CD4 + Th1 cells expressed higher levels of miR-19a-3p than CD4 + Th2 cells (Fig. 3A). Interestingly, the levels of miR-19a-3p decreased in both CD4 + Th1 and CD4 + Th2 cells over time, starting seven days after initial activation with anti-CD3/CD28 and polarization, and then reached levels slightly higher than those found in naïve CD4 + T cells. However, restimulation with anti-CD3/CD28 increased levels of miR-19a-3p in both CD4 + Th1 and CD4 + Th2 cells, similar to those observed on day 7 when the CD4 + T cells had reached their respective polarization. CD4 + Th1 cells continued to have higher levels of miR-19a-3p compared to CD4 + Th2 cells (Fig. 3B). Furthermore, restimulation with anti-CD3/CD28 of CD4 + Th1 and CD4 + Th2 cells induced an enrichment in the levels of miR-19a-3p measured in their respective supernatants after 24 h. A greater increase was observed in CD4 + Th1 cells compared to CD4 + Th2 cells, and this persisted until the end of the cell culture (Fig. 3C and 3D). The polarization of CD4 + Th1 and CD4 + Th2 cells remained stable throughout the cell culture period (Supplementary Fig. 3C-E). The culture of activated CD4 + T cells under non-polarizing conditions (Th0) resulted in a mixed Th1 and Th2 cytokine expression pattern, as expected (Supplementary Fig. 4A). The activation and restimulation with anti-CD3/CD28 induced a similar increase in the levels of miR-19a-3p, like those seen in polarized CD4 + Th1 and CD4 + Th2 cells (Supplementary Fig. 4B). This confirmed that activation and restimulation with anti-CD3/CD28 can boost the expression of miR-19a-3p in CD4 + T cells.

In summary, activating naïve CD4 + T cells with anti-CD3/CD28 under either non-polarizing (Th0) or polarizing (Th1 or Th2) conditions increased the levels of miR-19a-3p. After polarization, CD4 + Th1 cells exhibited higher levels of miR-19a-3p than CD4 + Th2 cells. Restimulating CD4 + Th1 and CD4 + Th2 cells with anti-CD3/CD28 resulted in increased levels of miR-19a-3p secreted into their respective supernatants, with CD4 + Th1 cells exhibiting the most significant rise in the levels of miR-19a-3p. Elevated levels of miR-19a-3p in CD4 + Th1 cells and their supernatants persisted throughout the cell culture period.

### Patients with metastatic HER2 + breast cancer and high levels of miR-19a-3p (good prognosis) had a higher percentage of circulating activated CD4 + T cells and NK cells compared to those with low levels of miR-19a-3p (poor prognosis)

**Fig. 4:**
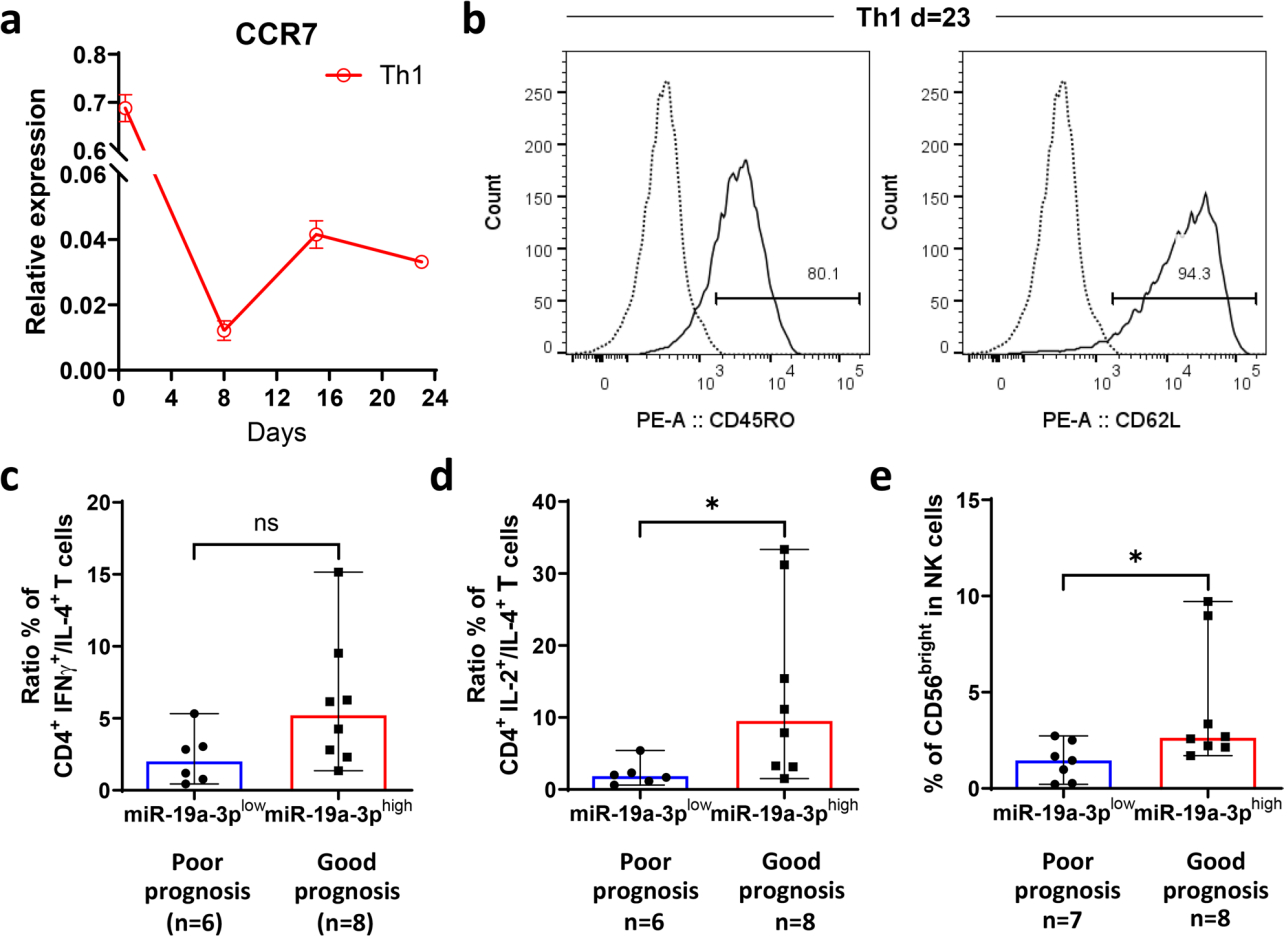
Central memory T (T_CM_) cell phenotype of CD4 + Th1 cells and immune cell populations in the peripheral blood of patients with metastatic HER2 + breast cancer. **a-b)** CD4 + Th1 cells expressing high levels of miR-19a-3p developed a central memory T (T_CM_) cell phenotype (CD45RO + CCR7 + CD62L +) by the end of the cell culture; **c-e)** in a small cohort of patients with metastatic HER2 + breast cancer, those with a good prognosis and high serum levels of miR-19a-3p showed a trend of higher percentage of CD4 + Th1 than CD4 + Th2 cells (ratio: IFN-γ + /IL-4 +), although not statistically significant (p = 0.08130) and activated CD4 + T and NK cells circulating in their blood. The results of panel **a** are shown as the mean ± SD of 3 technical replicates. The results in panel **b** are presented as the most representative of 3 technical replicates and two experimental replicates. The results in panels **c-e** are shown as the median with a 95% confidence interval (CI)(Available data from n = 15)

Examining the long-term in vitro culture (24 days) of CD4 + Th1 and CD4 + Th2 cells, we demonstrated that they maintained their polarized phenotype, as indicated by the expression of transcription factors and cytokines (Supplementary Fig. 3)** 3**). Additionally, CD4 + Th1 cells expressed and secreted higher levels of miR-19a-3p than CD4 + Th2 cells over time (Fig. 3). Because the development of the T_CM_ phenotype involves dynamic changes in the expression levels of memory markers (CD45RO + , CCR7 + , CD62L +) [59], we aimed to assess whether CD4 + Th1 cells developed a T_CM_ phenotype during long-term in vitro culture. The lymph node-homing CC-chemokine receptor 7 (CCR7), a chemokine receptor regulating the cell homing to secondary lymphoid organs, exhibited the typical expression kinetics pattern. Specifically, sorted naïve CD4 + T cells (CD45RA + CCR7 + CD62L +) had high levels of CCR7, which decreased after anti-CD3/CD28 activation over the 8 days of CD4 + T cell polarization as they differentiated into CD4 + Th1 cells. On day 9, CD4 + Th1 cells were restimulated with anti-CD3/CD28, and the levels of CCR7 increased and remained elevated over the following 14 days until day 23 (Fig. 4A). The persistent expression of CCR7 after effector phenotype differentiation characterizes the development of T_CM_ cells, as indicated by the expression of CCR7 along with CD45RO and CD62L [59]. Therefore, we assessed whether CD4 + Th1 cells developed a T_CM_ phenotype after 23 days of culture. Most CD4 + Th1 cells expressed both CD45RO (80.1%) and CD62L (94.3%) markers (Fig. 4B), confirming the development of the T_CM_ phenotype (CD45RO + CCR7 + CD62L +) [59–61] in CD4 + Th1 cells, which also express and secrete high levels of miR-19a-3p (Fig. 3). A similar pattern was observed with CD4 + Th2 cells (Supplementary Fig. 5).

The presence of CD4 + Th1 cells expressing IFN-γ in the TME is associated with beneficial clinical outcomes in patients with HER2 + breast cancer [16, 62] and other cancer types, including lung, colon, melanoma, and ovarian cancer [63]. T_CM_ cells can recirculate between the blood and lymphoid tissues [60]. Therefore, we aimed to assess whether the elevated levels of miR-19a-3p in the serum of patients with metastatic HER2 + breast cancer and a favorable prognosis were associated with the levels of CD4 + T cells expressing IFN-γ in the peripheral blood. In a small group (n = 15), we found that patients with metastatic HER2 + breast cancer and high serum levels of miR-19a-3p (indicating a good prognosis) had a trend of a higher ratio of CD4 + IFN-γ + /CD4 + IL-4 + T cells in their peripheral blood compared to those with low serum levels of miR-19a-3p (indicating a poor prognosis). However, this difference was not statistically significant (p = 0.0813) (Fig. 4C). Moreover, patients with metastatic HER2 + breast cancer and a good prognosis exhibited a higher proportion of CD4 + IL-2 + T cells relative to CD4 + IL-4 + T cells, along with an increased percentage of CD56^bright^ NK cells, in their peripheral blood compared to patients with a poor prognosis (Fig. 4D-E).

In summary, CD4 + Th1 cells that expressed and secreted high levels of miR-19a-3p developed a T_CM_ phenotype after 24 days of cell culture, and may contribute to the elevated levels of miR-19a-3p observed in the serum of patients with metastatic HER2 + breast cancer and a favorable prognosis. Additionally, a higher percentage of cells with anti-tumor functions, such as CD4 + IL-2 + T cells and CD56^bright^ NK cells [64], can be detected in the peripheral blood of patients with a favorable prognosis (elevated levels of miR-19a-3p) compared to those with a poor prognosis (low levels of miR-19a-3p).

## Discussion

The introduction of targeted anti-HER2 therapy with the monoclonal antibody trastuzumab (Herceptin) marked a significant advance in treating highly aggressive HER2-positive breast cancer. Although the evolution of trastuzumab with the generation of pertuzumab, and the antibody–drug conjugates trastuzumab deruxtecan (T-DXd) and trastuzumab emtansine (T-DM1), has led to improved survival rates for patients with HER2 + breast cancer [7], a substantial number of patients still develop treatment resistance [7, 65].

Besides blocking the oncogenic signals of amplified HER2 (PI3K/Akt and Ras/MAPK pathways), inhibiting cell cycle progression, reducing angiogenesis, and preventing the cleavage of the extracellular domain of HER2, trastuzumab mediates its anti-tumor activity by activating ADCC, which relies on effective innate and adaptive immunity [12]. An immunosuppressive TME can hinder ADCC [7]. Therefore, identifying predictive biomarkers that determine which patients are at risk of treatment failure and poor prognosis is essential.

Our previous study found that high levels of miR-19a-3p in the serum of patients with metastatic HER2 + breast cancer were associated with better clinical outcomes compared to those with low levels of miR-19a-3p [42]. In the current study, we aimed to understand the reasons behind the elevated levels of miR-19a-3p and identify the cells responsible for this phenomenon. We showed that trastuzumab binds to breast cancer cells at varying levels, depending on the HER2 expression levels and density on the cell membrane of MCF-7 (ER +), SKBR3 (HER2 +), and KPL-4 (HER2 +) cells. The lower levels of HER2 measured by trastuzumab compared to the anti-HER2 commercial antibody may result from differences in the affinity for the HER2 epitopes recognized by these antibodies. Additionally, the fluorophore PE generally produces a brighter signal and is less prone to photobleaching than the fluorophore FITC.

Effective NK cell functions induced by trastuzumab lead to efficient ADCC, which is associated with favorable clinical outcomes [66]. We demonstrated that the binding of trastuzumab to HER2 increased NK cell-mediated ADCC, which was associated with higher release of miR-19a-3p into the supernatants after the breast cancer cells were killed. This release correlated with the cellular levels of miR-19a-3p. It can be hypothesized that killing tumor cells that express high levels of miR-19a-3p may significantly contribute to the increased serum levels of miR-19a-3p. Additionally, larger tumor sizes with more tumor cells killed by NK-mediated ADCC may lead to a greater release of miR-19a-3p. Consequently, the elevated levels of miR-19a-3p observed in the serum of patients with metastatic HER2 + breast cancer may partly result from effective tumor cell killing by NK cell-mediated ADCC, reflecting a positive response to trastuzumab-based therapy associated with a favorable prognosis. Conversely, ineffective NK cell-mediated ADCC may be associated with decreased NK cell activation and reduced effector functions, such as lower tumor cell killing and IFN-γ production ability. This might explain the decreased levels of miR-19a-3p in the serum of patients with poor prognosis. Efficient ADCC is induced by antibody IgG Fc engagement with FcγRIIIA (CD16) on NK cells. Notably, nonsynonymous polymorphisms of FcγRIIIA influence the binding affinity of IgG Fc with FcγRIIIA and, accordingly, affect the ADCC triggering levels [67]. It is also important to note that combined chemotherapy may contribute to the killing of breast cancer cells and influence levels of miR-19a-3p in the serum of breast cancer patients.

Conflicting results have been reported regarding the benefits of adding trastuzumab to chemotherapy for patients with non-HER2-overexpressing breast cancer [55, 68–70]. We demonstrated that, consistent with previous reports [55], trastuzumab can also induce ADCC in non-HER2-overexpressing MCF-7 cells, which have a lower density of HER2 molecules on the cell membrane compared to the HER2-overexpressing SKBR3 and KPL-4 cells. It could be hypothesized that even a lower number of HER2 molecules on the breast cancer cell membrane (100,000 to 500,000 HER2 molecules on the cell membrane of score 1 + and 2 + breast cancer cells versus over two million on score 3 + cells) [71, 72] could be enough to elicit an effective NK cell-mediated ADCC. Therefore, our findings may support future research into whether levels of miR-19a-3p in patients with non-HER2-overexpressing breast cancer can help identify those who would benefit from trastuzumab treatment.

It is important to note that, in addition to NK cells, monocyte/macrophage cells expressing activating FcγRs (CD64, CD32a, and CD16) also contribute to the antitumor efficacy of trastuzumab through antibody-dependent cellular phagocytosis (ADCP) and ADCC [7, 73]. It was reported that miR-19a-3p regulates the polarization of tumor-associated macrophages (TAMs) into the M1 phenotype [74, 75]. In addition to ADCP and ADCC, monocyte/macrophage cells may also secrete miR-19a-3p, thereby influencing its levels in breast cancer patients with a favorable prognosis, similar to CD4 + Th1 cells. Since these mechanisms are not directly tested in the current study, they remain hypothetical and should be validated in future work. A larger patient cohort in a prospective study, a comparison between homogeneous and controlled groups, and in vivo functional validation are necessary to address the limitations of our retrospective research and enable further investigations into different immune cell contributions to miR-19a-3p.

Since it is involved in the polarization of immune cells that play a role in immune responses, such as CD4 + Th1 cells and M1 macrophages [74, 75], miR-19a-3p may have a role in antitumor immunity. The IL-4R/STAT6-dependent pathway induces the downregulation of the miR-17–92 cluster in splenocytes from C57BL/6 mice bearing B16 subcutaneous tumors and in T lymphocytes from glioblastoma multiforme (GBM) patients. Conversely, T cells from transgenic mice overexpressing the miR-17–92 cluster show an enhanced type-1 phenotype, characterized by increased production of IFN-γ and IL-2 [39]. The miR-19a-3p-mediated downregulation of the two targets, PTEN and BCL2L11, helps sustain proliferation, T helper cell cytokine secretion, and resistance to apoptosis in T cells [76–79].

Because trastuzumab can enhance Th1 cell-mediated antitumor immunity [16, 58, 62], and miR-19a-3p regulates T cell functions, we investigated the association between the levels of miR-19a-3p and immune responses in HER2 + breast cancer. Effective interaction among various cell components of innate and adaptive immunity is crucial for fully activating NK cells, which leads to effective ADCC and anti-tumor responses [44, 58, 80–85]. CD4 + Th1-mediated functions are essential for effective anti-tumor responses, as they promote the proliferation and cytotoxic activity of CD8 + CTLs and NK cells. Activated NK cells produce IFN-γ and T cell-recruiting chemokines, including IL-8, MIP-1α, MIP-1β, MCP-1, and RANTES [9, 10, 86]. IFN-γ promotes CD4 + Th1 cell polarization [87], and the levels of CD4 + Th1 cells infiltrating the tumor microenvironment are associated with favorable clinical outcomes [88–91]. High levels of tumor-infiltrating lymphocytes (TILs) in patients with metastatic HER2 + breast cancer treated with pertuzumab, trastuzumab, and taxane-based chemotherapy as first-line therapy are associated with a better prognosis [92]. We observed that CD4 + Th1 cells expressed and secreted higher levels of miR-19a-3p compared to CD4 + Th2 cells. Additionally, the expression and secretion levels of miR-19a-3p increased after each anti-CD3/CD28 restimulation, helping to maintain sustained levels over time. The anti-CD3/CD28 restimulation mimics the stimulation that CD4 + Th1 cells receive from antigen-presenting cells (APCs), such as TAAs presented by dendritic cells in the TME. Tumor cells killed by NK cell-mediated ADCC serve as a source of TAAs that are presented by APCs to support the stimulation of CD4 + Th1 cells, thereby maintaining high levels of miR-19a-3p. The elevated levels of miR-19a-3p expressed and secreted by CD4 + Th1 cells can potentially serve as a biomarker for CD4 + Th1 cell activation and effector functions, such as IFN-γ production, suggesting an immunocompetent TME necessary for an effective NK cell-mediated ADCC.

T_CM_ cells traffic to lymphoid tissues, where they produce effector cytokines and differentiate into effector memory T (T_EM_) cells in response to antigens [59, 60]. Notably, we observed that CD4 + Th1 cells developed a T_CM_ phenotype (CD45RO + CCR7 + CD62L +) after 24 days of in vitro culture and expressed and secreted high levels of miR-19a-3p following anti-CD3/CD28 restimulation. This suggests that increased levels of IFN-γ and TAAs presented by APCs after effective NK cell-mediated ADCC can sustain the stimulation of CD4 + Th1 T_CM_ cells in the draining lymph nodes, prompting them to express and secrete miR-19a-3p and eventually differentiate into T_EM_. This could help explain the elevated serum levels of miR-19a-3p found in patients with metastatic HER2 + breast cancer who have a better prognosis.

High levels of CD4 + Th1 cells that express IFN-γ in the blood are associated with a positive response to cancer [93, 94]. In a small group of patients, we observed that those with metastatic HER2 + breast cancer and high serum levels of miR-19a-3p (indicating a good prognosis) exhibited a trend of higher levels of activated CD4 + T cells secreting IL-2 and IFN-γ, as well as NK cells, in their blood compared to patients with low levels of miR-19a-3p (indicating a poor prognosis). This suggests that elevated serum levels of miR-19a-3p are associated with increased circulating immune cells that have anti-tumor functions, reflecting the activation of an anti-tumor immune response responsible for the favorable prognosis. We acknowledge that the small cohort size limits the statistical power of our retrospective study. This limitation is particularly evident in the comparison of the CD4 + IFN-γ + /IL-4 + T cell ratio, where a non-statistically significant difference was observed, making it difficult to draw definitive conclusions. Future prospective studies with larger patient cohorts and control groups are necessary to validate these findings. 

**Fig. 5:**
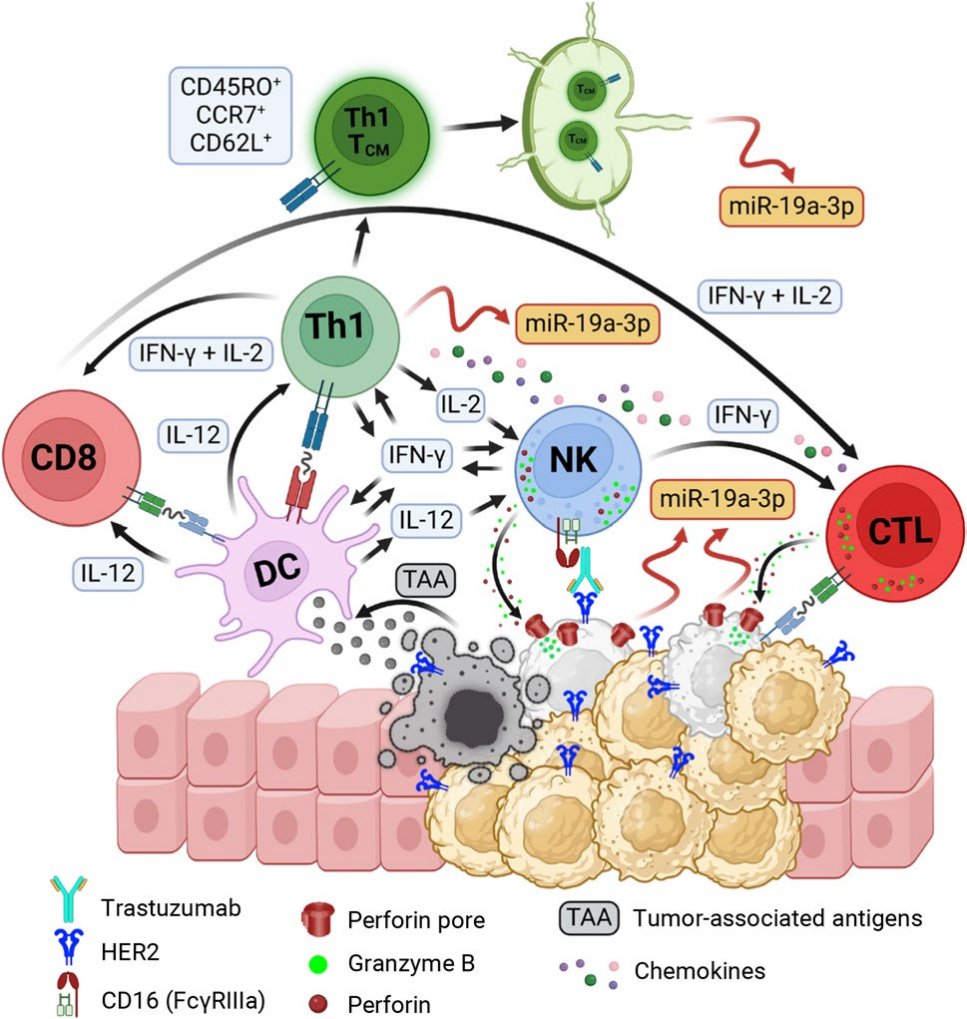
Proposed model of TME of HER2 + breast cancer modulated by NK cell-mediated ADCC. The proposed model is based on findings from our current and previous work. Further studies are needed to validate and confirm our hypothetical model. Trastuzumab binds to HER2 + cells and engages CD16 on NK cells, activating them and inducing ADCC. This leads to perforin/granzyme B-mediated apoptosis and death of HER2 + breast cancer cells, as well as the release of miR-19a-3p into the TME and eventually into the bloodstream. ADCCalso stimulates the production and secretion of IFN-γ and T cell-recruiting chemokines from NK cells, which activate DCs, promote their migration to draining lymph nodes, and induce migration of lymphocytes into the TME. This process enables TAA cross-presentation to T cells (both CD4 + and CD8 +) and triggers the production and secretion of IL-12 by DCs. IL-12 and IFN-γ promote the differentiation of CD4 + and CD8 + T cells into CD4 + Th1 cells and CD8 + CTLs and enhance the cytotoxic activity of CD8 + CTLs and NK cells. IL-2 promotes the proliferation of CD4 + Th1 cells, CD8 + CTLs, and NK cells, while IFN-γ from CD4 + Th1 and NK cells further activates DCs (enhancing TAA cross-presentation and cytokine release). The activation and differentiation of CD4 + Th1 cells increase the expression and secretion of miR-19a-3p into the TME and bloodstream. Activated CD8 + CTLs infiltrate tumor tissue and work with NK cells to kill tumor cells, further increasing the release of miR-19a-3p. Additionally, CD4 + Th1 cells differentiate into CD4 + Th1 T_CM_ cells (CD45RO + CCR7 + CD62L +). TAAs from the TME and presented by DCs can maintain the expression and secretion of miR-19a-3p by stimulating CD4 + Th1 T_CM_ cells in draining lymph nodes and contributing to higher serum levels of miR-19a-3p. (This figure was created in **BioRender**)

Based on the results from our current and past studies, we propose a hypothetical model of the TME in HER2 + breast cancer to explain the potential mechanisms behind the favorable prognosis of patients with HER2 + breast cancer, which is responsible for the high serum levels of miR-19a-3p (Fig. 5). Trastuzumab induces NK cell-mediated ADCC, and activated NK cells produce IFN-γ and T cell-recruiting chemokines, including IL-8, MIP-1α, MIP-1β, MCP-1, and RANTES, promoting migration into the TME [9, 10, 86]. IFN-γ promotes the activation, maturation, and antigen-presenting functions of DCs [95–97] and the differentiation of naïve CD4 + T cells into CD4 + Th1 effector cells [80]. The IFN-γ from CD4 + Th1 cells further activates DCs, promotes TAA cross-presentation, and stimulates the production of IL-12 by DCs [96, 98]. IL-12 promotes CD4 + Th1 cell polarization along with IFN-γ [99], enhances the cytotoxic activity and IFN-γ production in NK cells [9, 21, 44, 86], and induces the generation and activation of CD8 + CTLs in combination with IFN-γ [100, 101]. Finally, IL-2 from CD4 + Th1 cells, in combination with IFN-γ, promotes cell proliferation and cytotoxic activity of CD8 + CTLs and NK cells working together to kill tumor cells [9, 21, 102]. An ineffective NK cell-mediated ADCC can be associated with decreased IFN-γ and chemokine production by NK cells, as well as reduced tumor cell killing. Notably, an immunosuppressive TME, characterized by IL-10, TGF-β, PD-L1, MDSCs, and Treg, can render NK cells dysfunctional, thereby hindering effective NK cell-mediated ADCC [103]. This results in a diminished ability to elicit an effective CD4 + Th1-mediated anti-tumor immune response, which may explain the poor prognosis of patients, characterized by low serum levels of miR-19a-3p. Since trastuzumab can upregulate PD-L1 through IFN-γ [104], the combination with immune checkpoint inhibitor (ICI) therapy can enhance NK cell-mediated ADCC [105, 106].

In conclusion, our study showed that NK cell-mediated ADCC and the activation of CD4 + Th1 cells can lead to the release of high levels of miR-19a-3p into cell supernatants. Since increased levels of miR-19a-3p in the serum of patients with metastatic HER2 + breast cancer treated with trastuzumab were associated with favorable clinical outcomes, we believe that miR-19a-3p could serve as a circulating blood biomarker to identify patients with effective anti-tumor immune responses activated by trastuzumab, which involves NK cell-mediated ADCC and the activation of CD4 + Th1 cells. The small cohort size (n = 15) limits the statistical power of our retrospective study. Future prospective studies are necessary to determine the predictive value of serum miR-19a-3p and to enhance its clinical usefulness as a biomarker.

As future developments, our findings could potentially be applied to study the activation of ADCC and CD4 + Th1-mediated immune responses induced by other antibodies alone or in combination with chemotherapy and ICI therapy. This includes the anti-EGFR cetuximab in metastatic colon cancer, head and neck cancer (HNSCC), triple-negative breast cancer (TNBC), non-small cell lung cancer (NSCLC), the anti-CD20 rituximab in chronic lymphocytic leukemia (CLL) and non-Hodgkin lymphoma (NHL), and the anti-PD-L1 avelumab in pancreatic cancer, HNSCC, and NSCLC [9, 107–110]. The potential applications of our findings may also include chimeric antigen receptor-modified T or NK cells (CAR-T or CAR-NK) as anti-cancer therapies, since CAR engagement with specific targets can induce higher levels of IFN-γ than native T and NK cells [111]. Additionally, using in vitro assembloids that replicate the TME of HER2 + breast cancer, as shown in Fig. 5, using either miR-19a-3p-expressing or non-expressing modified cells (including HER2 + tumor cells, NK cells, CD8 + CTL, CD4 + Th1 cells, and macrophages), can provide further insights into the cellular origin of circulating miR-19a-3p and the macrophage-mediated ADCC and ADCP [73, 112, 113]. Finally, a Droplet Digital PCR (ddPCR) assay could be developed for standardized absolute quantification of serum or plasma miR-19a-3p in clinical settings [114].

## Supplementary Information

Below is the link to the electronic supplementary material.

Supplementary Material 1

Supplementary Material 2

## Data Availability

No datasets were generated or analysed during the current study.
