# Supplementary material for: High levels of circulating miR-19a-3p in patients with metastatic HER2 + breast cancer are associated with a favorable prognosis and anti-tumor immune responses
Source: Breast Cancer Res. 2026 Jan 26;28:3. doi: 10.1186/s13058-025-02174-8 (PMC12833941; doi:10.1186/s13058-025-02174-8)

Supplementary Figure 1

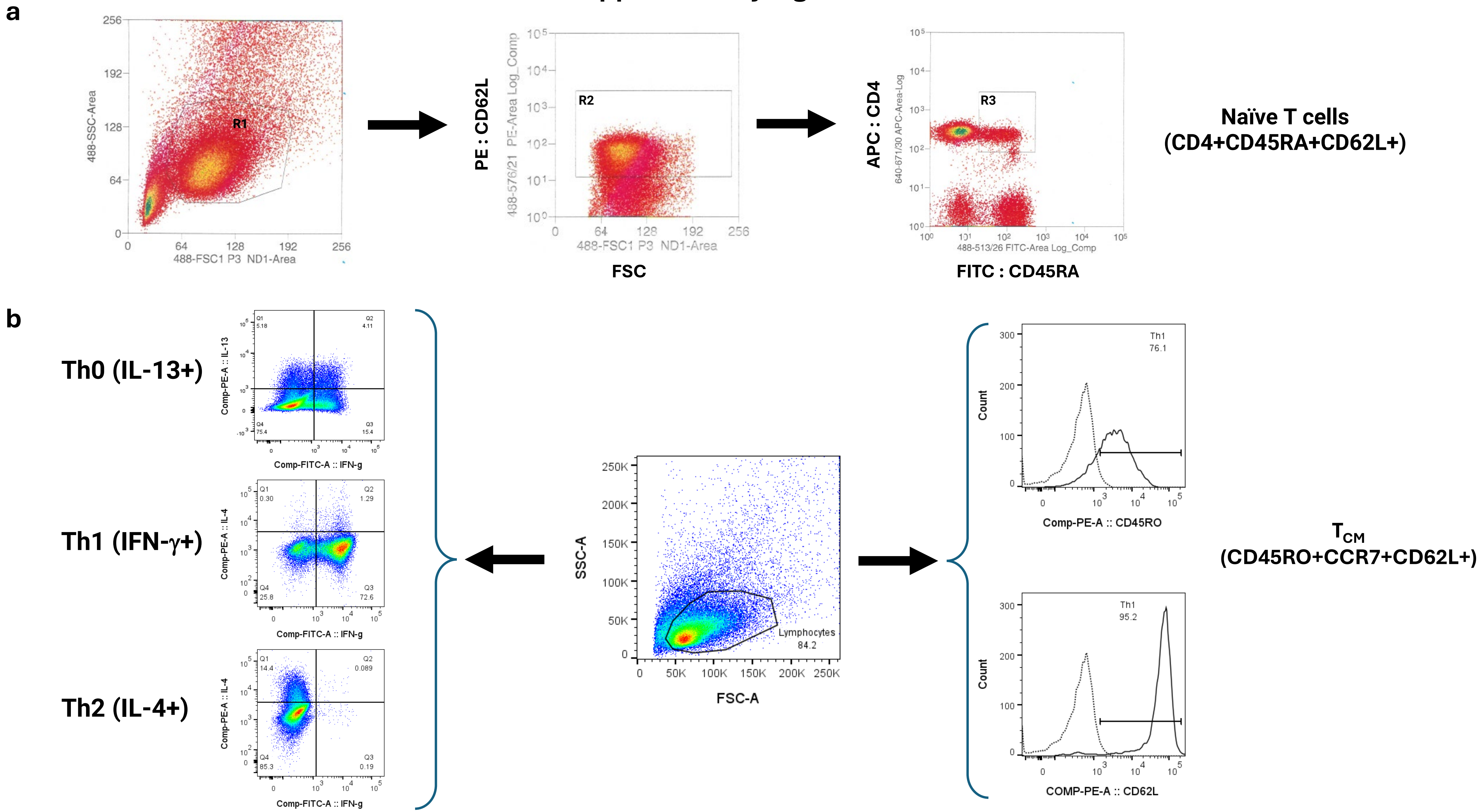

Supplementary Figure 1

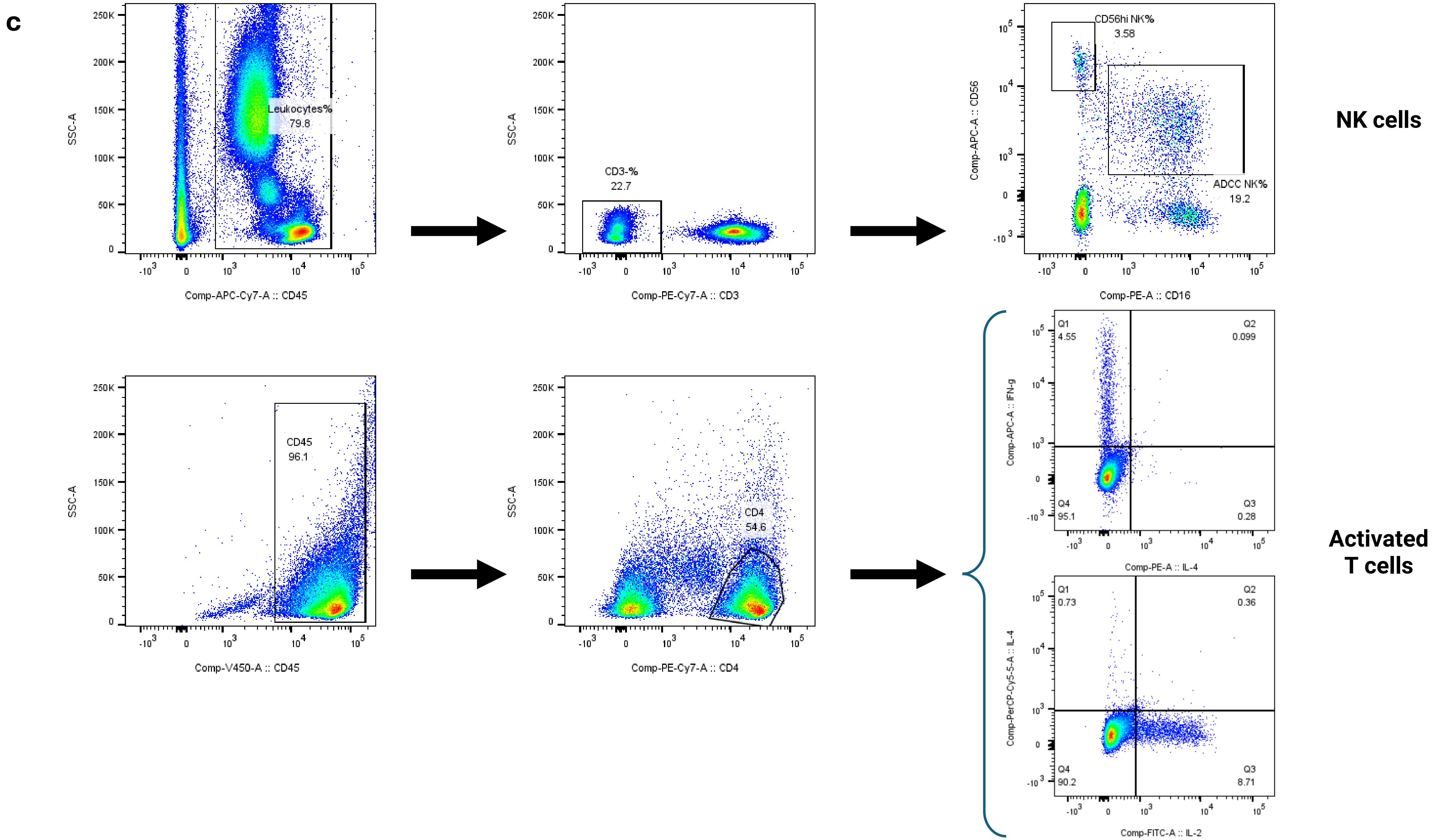

Supplementary Figure 2

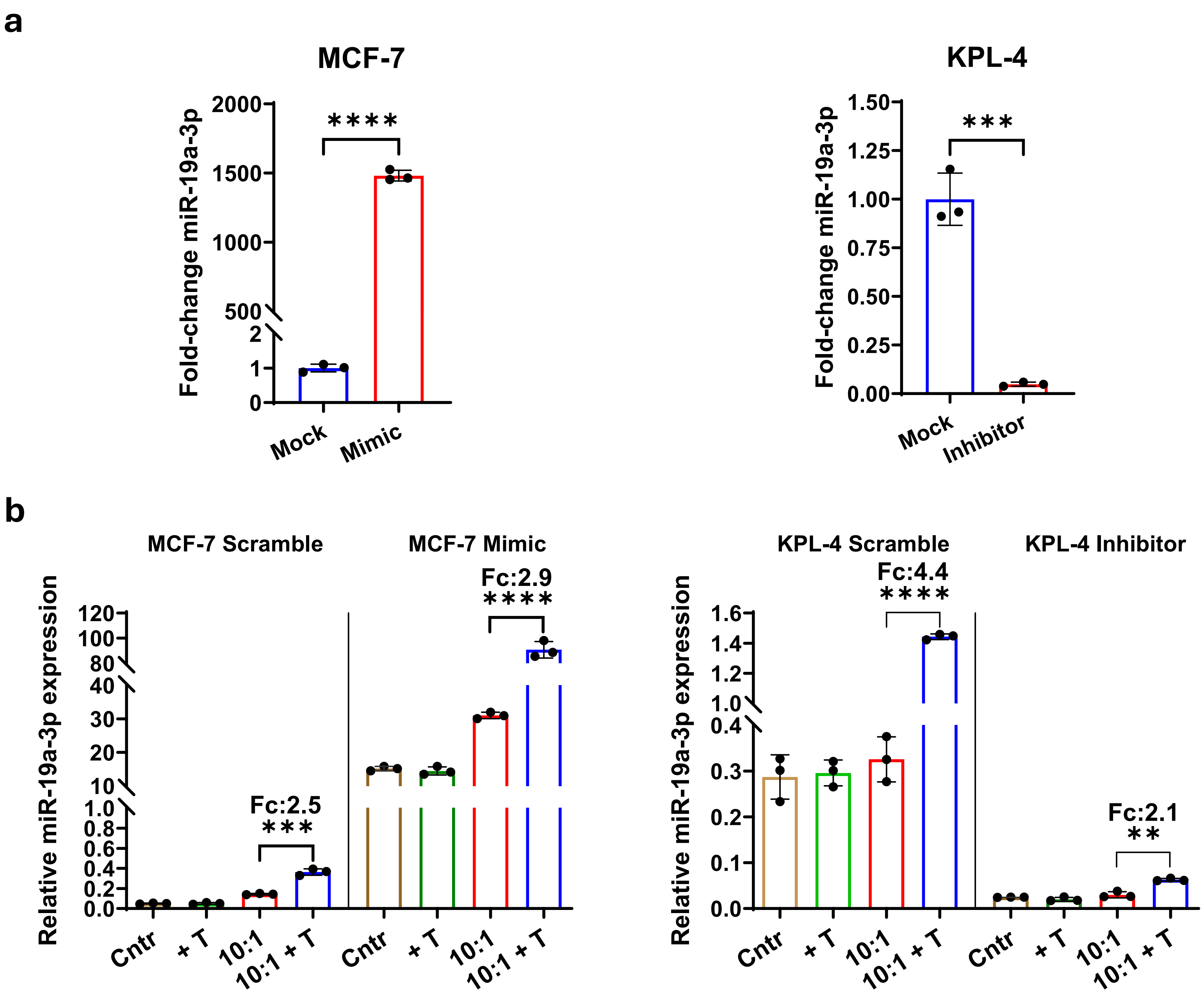

Supplementary Figure 3

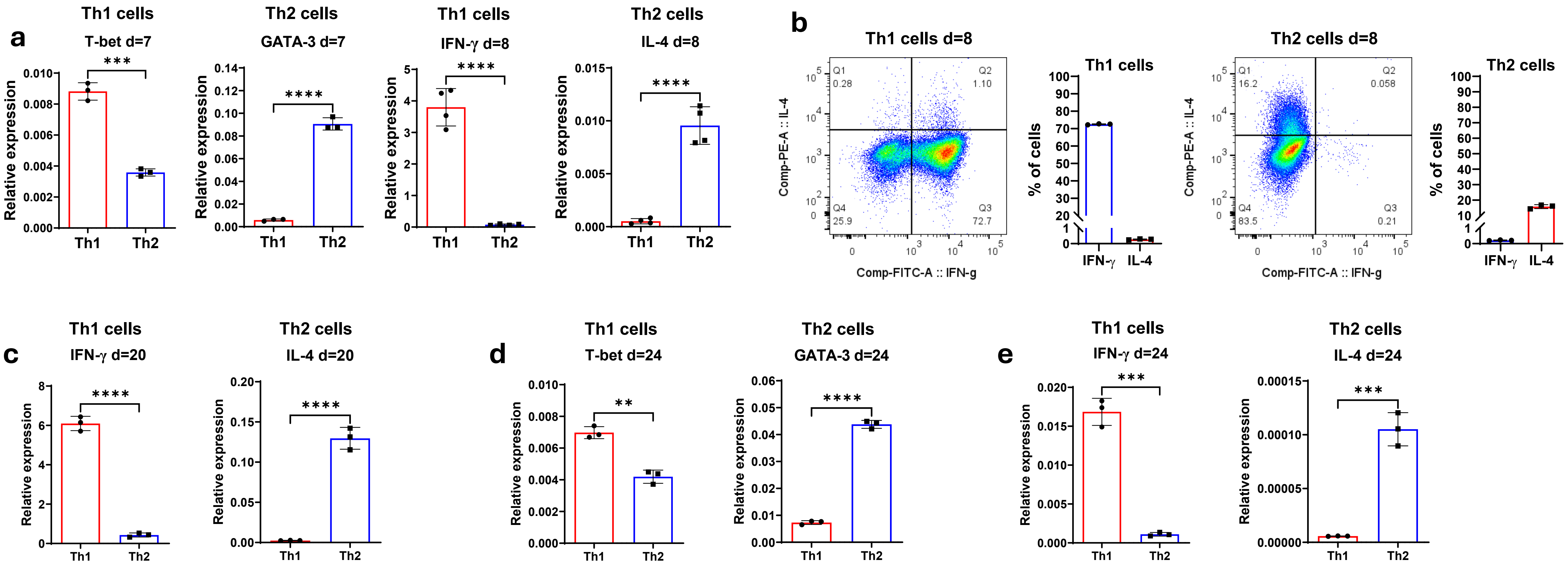

Supplementary Figure 4

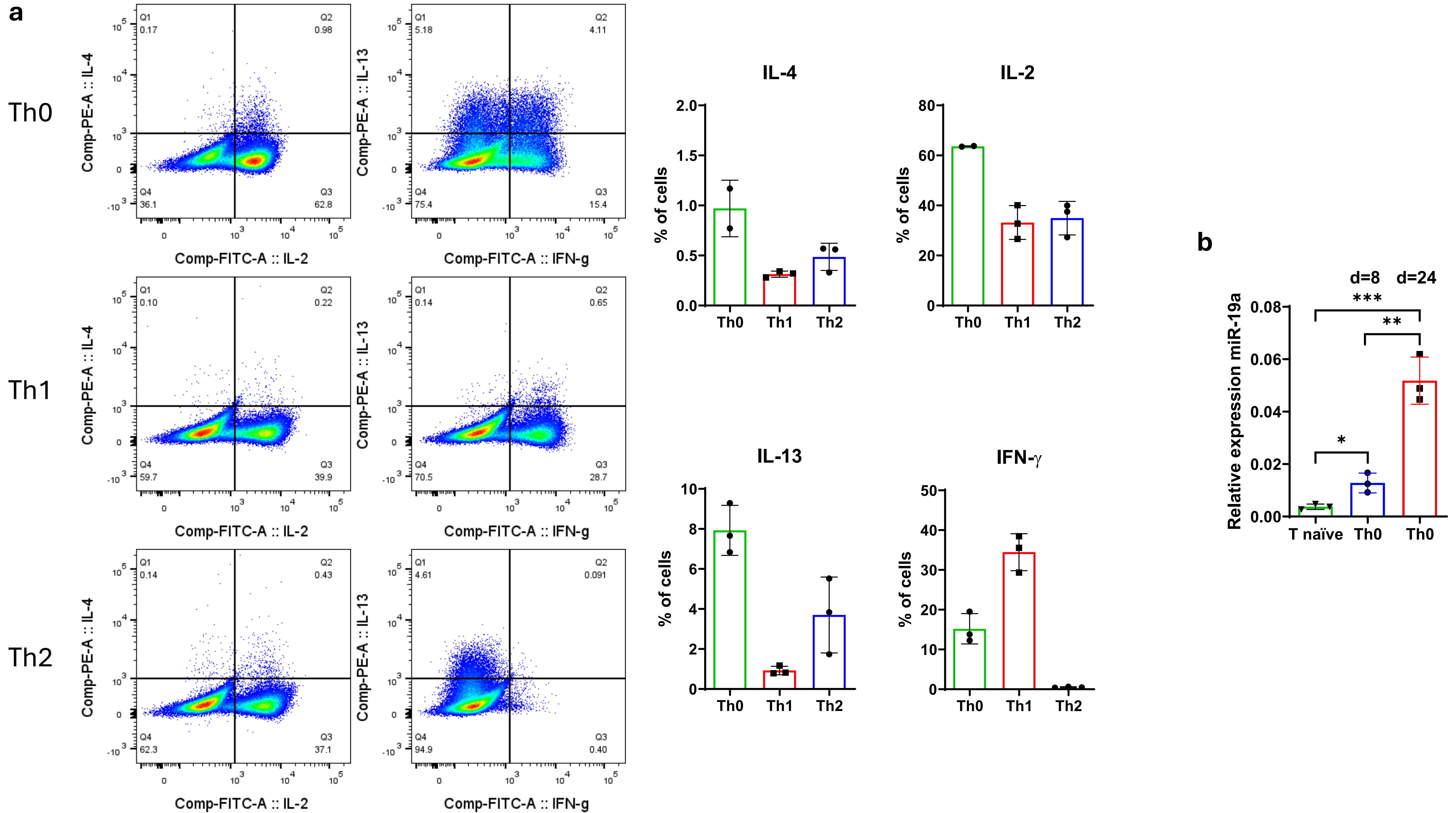

Supplementary Figure 5

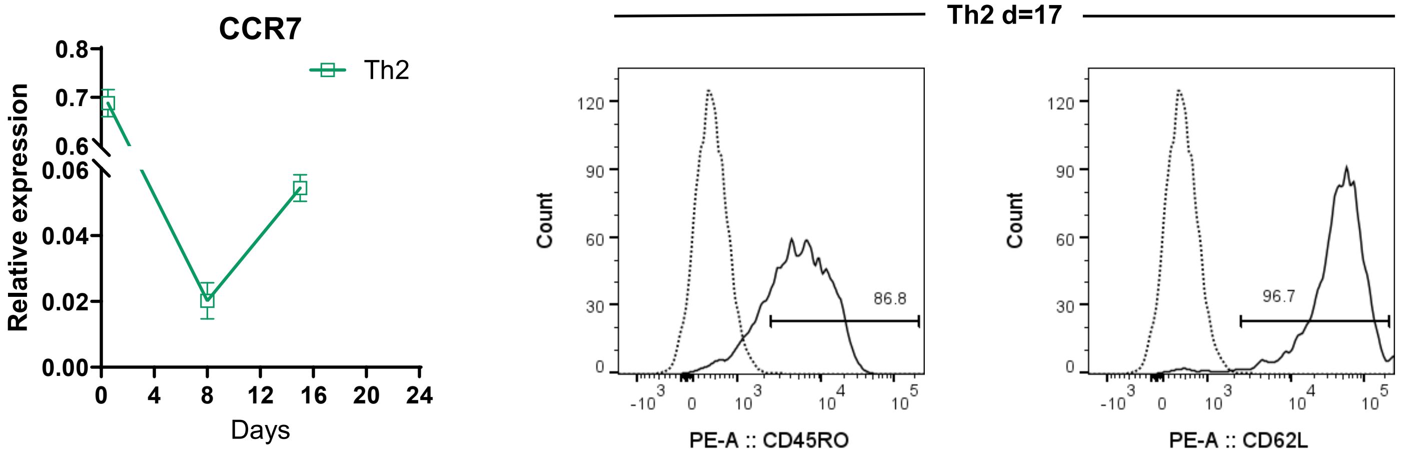

Supplement: Supplementary file 1 — Supplementary Material 1 [file 13058_2025_2174_MOESM1_ESM.pdf]
