# Supplementary material for: High levels of circulating miR-19a-3p in patients with metastatic HER2 + breast cancer are associated with a favorable prognosis and anti-tumor immune responses
Source: Breast Cancer Res. 2026 Jan 26;28:3. doi: 10.1186/s13058-025-02174-8 (PMC12833941; doi:10.1186/s13058-025-02174-8)
Supplement: Supplementary file 2 — Supplementary Material 2 [file 13058_2025_2174_MOESM2_ESM.docx]

**Supplementary figure legends**

**Supplementary Figure 1.** **Flow cytometry gating strategy**. **a**) PBMCs from normal individuals were sorted to isolate naïve T cells (CD4+CD45RA+CD62L+); **b**) CD4+ T cells cultured under both non-polarizing and polarizing conditions were investigated for their Th0, Th1, Th2, and TCM cell phenotype; **c**) breast cancer patients’ PBMCs were stained with anti-CD45 to identify leukocytes. The CD3-negative population was used to identify NK cells (CD56+CD16+), while the CD4-positive population was used to identify activated T cells.

**Supplementary Figure 2.** **NK cell-mediated ADCC of transfected MCF-7 and KLP-4 cells**. **a**) Transfection of MCF-7 and KPL-4 cells with miR-19a-3p mimic and inhibitor resulted in increased and decreased cellular levels of miR-19a-3p, respectively; **b**) NK cell-mediated ADCC caused different amounts of miR-19a-3p to be released into cell supernatants depending on the levels of miR-19a-3p in MCF-7 and KPL-4 cells. The results are shown as the mean ± SD of 3 technical replicates.

**Supplementary Figure 3.** **CD4+** **Th1 and CD4+** **Th2 phenotypes**. **a**) Polarization of CD4+ Th1 and CD4+ Th2 cells from freshly isolated naïve CD4+ T cells (CD45RA+CCR7+CD62L+), with their respective phenotypes assessed by the expression levels of specific polarization markers, including transcription factors (T-bet for CD4+ Th1 cells and GATA-3 for CD4+ Th2 cells), and cytokines (IFN-γ for CD4+ Th1 cells and IL-4 for CD4+ Th2 cells) measured by RT-qPCR. Cytokine expression levels were measured after PMA/ionomycin stimulation; **b**) expression levels of IFN-γ and IL-4 in Th1 and Th2 cells measured by flow cytometry after PMA/ionomycin stimulation; **c**) expression levels of Th1 and Th2 cytokines (IFN-γ and IL-4) after the PMA/ionomycin stimulation at d=20; **d-e**) basal expression levels of Th1 and Th2 cytokines (IFN-γ and IL-4) and transcription factors (T-bet and GATA-3) measured at the end of the cell culture by RT-qPCR. The results are shown as the mean ± SD of 3 technical replicates, except for IFN-γ and IL-4 in panel **a** (4 technical replicates).

**Supplementary Figure 4.** **Expression levels of miR-19a-3p in non-polarized CD4+ Th0 cells**. **a**) CD4+ T cells cultured under non-polarizing (Th0) conditions show a mixed cytokine expression profile, including both Th1 (IFN-γ+) and Th2 (IL-4+ and IL-13) cell cytokines (d=8). IL-2 was used as an activation marker; **b**) Th0 cells express increased levels of miR-19a-3p compared to naïve T cells at d=8, and restimulation with anti-CD3/CD28 further boosted the expression levels after 24 hours at d=24. The results are shown as the mean ± SD of 3 technical replicates, except for IL-4 and IL-2 in Th0 in panel **a** (2 technical replicates)

**Supplementary Figure 5.** **T_CM_ phenotype of** **CD4+** **Th2 cells**. CD4+ Th2 cells acquire the T_CM_ phenotype (CD45RO+CCR7+CD62L+) like CD4+ Th1 cells. The results of CCR7 expression are shown as the mean ± SD of 3 technical replicates.
